# Open Source Software to Control Bioflo Bioreactors

**DOI:** 10.1371/journal.pone.0092108

**Published:** 2014-03-25

**Authors:** David A. Burdge, Igor G. L. Libourel

**Affiliations:** 1 Biotechnology Institute, University of Minnesota, Saint Paul, Minnesota, United States of America; 2 Department of Plant Biology, University of Minnesota, Saint Paul, Minnesota, United States of America; CSIR Institute of Genomics and Integrative Biology, India

## Abstract

Bioreactors are designed to support highly controlled environments for growth of tissues, cell cultures or microbial cultures. A variety of bioreactors are commercially available, often including sophisticated software to enhance the functionality of the bioreactor. However, experiments that the bioreactor hardware can support, but that were not envisioned during the software design cannot be performed without developing custom software. In addition, support for third party or custom designed auxiliary hardware is often sparse or absent. This work presents flexible open source freeware for the control of bioreactors of the Bioflo product family. The functionality of the software includes setpoint control, data logging, and protocol execution. Auxiliary hardware can be easily integrated and controlled through an integrated plugin interface without altering existing software. Simple experimental protocols can be entered as a CSV scripting file, and a Python-based protocol execution model is included for more demanding conditional experimental control. The software was designed to be a more flexible and free open source alternative to the commercially available solution. The source code and various auxiliary hardware plugins are publicly available for download from https://github.com/LibourelLab/BiofloSoftware. In addition to the source code, the software was compiled and packaged as a self-installing file for 32 and 64 bit windows operating systems. The compiled software will be able to control a Bioflo system, and will not require the installation of LabVIEW.

## Introduction

Bioreactor systems are complex devices designed for the sophisticated control of culture environments. Commercially available culturing systems have integrated closed-loop control of process variables such as pH, dissolved oxygen, and temperature. In industry, bioreactors are commonly used to facilitate substrate to product conversion processes in fermentation and cell culturing. Continuous and rigid control of the environment is important to optimize product yields of chemical by-product or total cell biomass [1], [2]. In a research setting bioreactors are used for experiments that require careful control of environmental parameters. In such setting, the automation of user defined experimental protocols if often required in addition to maintaining a static environment. To fulfill the need for execution of customized protocols, many fermentation systems are coupled to a personal computer running commercially available bioreactor control software [3]. In addition to enabling protocol execution, software provides data logging and visualization capabilities of reactor parameters. However, many commercial software packages lack the ability to integrate auxiliary hardware, and cannot implement the custom built control algorithms that are needed for more sophisticated experiments [4]. Due to these short comings, researchers have resorted to developing custom built reactor control software, including algorithms that handle axillary hardware [5]. The steep learning curve of most programming languages presents a significant hurdle for many scientist interested in developing software that implements custom algorithms. In addition, maintenance of software and adaptation to the frequently changing requirements of researchers constitutes a significant investment and long term commitment. To reduce the learning curve associated with program development using text based languages, a visual programming environment named LabVIEW emerged as a development environment of choice for bioprocess control and automation [4]. As a result LabVIEW has been used to control many bioreactor systems including: fed-batch [6], hollow-fiber [7], air lift [8], and catalytic packed-bed [9] reactors. While many of these systems are composed of an assortment of hardware [10], some researchers were able to utilize the hardware capabilities of commercial bioreactor systems to develop custom control systems with the addition of auxiliary hardware [11]. Given that many control processes require significant execution time, the multitasking capability of LabVIEW is essential in maintaining a responsive control interface. Multitasking is accompanied with an assortment of potential timing conflicts that need to be navigated. Most of the potential complications can be avoided by implementing existing software architectural concepts. These include maintaining scalability [12], and proper decoupling of asynchronous processes. Inadequate handling of asynchronous processes can cause loss of data [13] or the loss of control signals [14]. An effective architecture for maintaining scalability is the state machine (SM). Although SMs are effective for small to medium sized application, larger applications typically utilize queued SMs (QSM) to facilitate complex access control of resources [14]. A QSM can be accompanied with a producer-consumer pattern (QSM-PC) to decouple asynchronous processes. Examples of QSM-PC implementation are the decoupling of data acquisition from data analysis [15], hardware control from hardware communication [16], and user interactions from code execution [13].

This work describes the flexible, open source software package that was developed to control Bioflo family bioreactors using LabVIEW. The software is capable of controlling, monitoring, data logging, and protocol execution. By utilizing the supervisory control capability packaged with the Bioflo bioreactor, complex control systems can be developed without modification of the hardware. Auxiliary hardware is not utilized in this work, but the software was designed to facilitate easy integration of custom hardware through a plugin interface and several example plugins are available for download from the project page. This work expands on the software development previously mentioned by presenting methods to control the distribution of shared hardware and software resources. The software is capable of simultaneously controlling multiple reactors through a single interface that provides a stable and customizable control system for the Bioflo systems. This was demonstrated by concurrently executing independent control systems for the Bioflo3000 and Bioflo110 system.

## Materials and Methods

### Bioflo110 Benchtop Bioreactor

The Bioflo110 (Eppendorf AG, Hamburg/Germany) is a modular fermentation system for cell culture and fermentation systems. The 1.3 liter glass vessel is equipped with a motor driven impeller, glass pH probe, Clark-type dissolved oxygen (DO) probe, RTD temperature sensor, level probe, gas sparing coil, heat blanket, condensing exhaust port and various liquid inlets and outlets. The vessels hardware and sensors interface with stacked control modules that communicate with a Primary Control Unit (PCU) via a daisy chained RS-485 control bus. The PCU is capable of controlling up to four separate vessels with a maximum of sixteen control modules in total. The PCU provides a user interface to the control system of each vessel. The vessel control architecture is separated into loops, each including a sensor and a control element. Examples of such control loops are temperature, agitation speed, pH and DO control. The pump loops can be configured to operate in response to the liquid level inside the vessel measured with a level probe. All other control loops can be configured to utilize an integrated PID (proportional-integral-derivative controller) algorithm to coerce the sensor value to the user defined setpoint using the control element.

### Reading and writing the Bioflo110

The bioflo110 was connected to a supervisory computer through a female DB-25 connector located on the rear of the primary control unit (PCU). The DB-25 connector contains pin outs for both RS-422 and RS-232 physical communication layers (Guide to Operations). Because the added complexity of the RS-422's master-slave functionality was not needed, the RS-232 standard was chosen. A male DB-25 to male DB-9 patch cable was custom made, and connected to an RS-232 to USB conversion cable that was interfaced with the supervisory computer (Supplement S1). Two equivalent communication protocols can be used to communicate with the PCU: ModBUS and AFS. The AFS protocol was chosen for use in this software because other members of the BioFlo reactor family support this protocol. To read control loop properties a ‘request message’ is sent to the PCU. A list of request message types, formats and responses is included in supplement S1. The PCU responds to a request message with a response header and the requested information in the message format. The setpoints and outputs of the control loops are changed by sending a ‘command message’ to the PCU. A command message uses the format header and the command information in the message format (Supplement S1). The PCU responds to command messages by acknowledging the change, and the reactor vessel the change applied to (multidrop number).

### LabVIEW software

LabVIEW is a graphical programming language designed for hardware automation. Programs in LabVIEW are called virtual instruments (VIs) and integrate a graphical user interface (GUI) with the development of code. A VI contains three components: a front panel, a block diagram, and a connector panel. The front panel serves as the GUI which contains elements referred to as controls and indicators. Controls such as buttons, sliders, and text boxes, allow the user to manipulate the value of data used during code execution. Data values are displayed on the front panel with indicators such as graphs, indicator lights, and gauges. Controls and indicators appear as terminals on the block diagram where code development takes place. The block diagram contains other elements called subVIs, functions, constants, structures, and wires. Data from control terminals and constants flows from left to right through wires to functions and subVI input nodes where data operations are performed. Function and subVI output nodes wire data from operations to indicator terminals for display on the front panel. SubVIs, which are VIs executed on the block diagram of another VI, connect data from input and output nodes to controls and indicators through the connector pane. Block diagram structures, such as while loops, control program execution and can reroute the data flowing through wires allowing for more complex functionality. Due to LabVIEWs native multitasking capabilities, execution of parallel processes, such as data flowing through wires, occurs concurrently.

## Results and Discussion

Following installation using the self-installer executable, the bioreactor that is connected to the computer as described in the material and methods can be controlled by the bioreactor software. Data-logging starts immediately upon selecting a control system name, a plugins root folder, and a data logging folder which are prompted for at startup. If the data logging file already exists, new data will be appended. A viewer for the logged data, as well as a detailed description of the installation procedure and operation manual of the software are included as downloads on the project site.

### Software Design

To create stable and scalable control system software, functionality was distributed amongst independent subprograms (VIs) here referred to as actors. Each actor fulfills a specific function, such as hardware communication or protocol execution, and contains a QSM-PC structure to decouple asynchronous processes. Control systems and their actors are launched, managed and closed by the manager VI. The manager VI is capable of concurrently operating multiple control systems, which can be added or removed by the user on the fly. Interaction with actors occurs through a subpanel display on the manager VI front panel which integrates the program into a single user interface.

Control system actors are loaded into memory and launched dynamically by the manager VI through an integrated plugin interface. The plugin interface automates the interaction between the manager VI and control system actors. Actors, formatted according to the plugin interface, are compiled into packaged libraries called plugins. These plugins can be developed independently of the manager VI, and are intended to expand the program functionality to include auxiliary hardware without modification of the manager VI. Plugins associated with a control system are localized within the same PC directory on the supervisory computer. When adding a control system, the user is prompted to select the PC directory to load and launch plugins from. By selecting different control system PC directories, unique control systems can operate simultaneously. Plugins can be created in the LabVIEW development environment using the plugin interface which can be downloaded from https://github.com/LibourelLab/BiofloSoftware along with several plugin examples.

Communication between actors within a control system occurs through a shared data resource. Access to the data resource is given to each actor within the control system when launched. Actors populate the data resource with state information and periodically update the values with current values. Each actor reads from, and writes to, the shared data resource, allowing for communication between actors.

To maintain a scalable control system, data logging operations of an actor is performed by the actor itself. This provides a data logging method that can be reused for a scalable number of actors. Each actor logs data in a binary file format that is compact, secure and appendable. Upon adding a control system each actor queries for an existing data file that is associated with itself. If an actor finds an existing file the most current data values are read from the file and written to the shared data resource. This enables continuation of interrupted experiments with the same state information. Data files are periodically appended with state information by the actor and closed with the actor when the control system is removed. Data files can be browsed using a bundled data viewer VI or loaded into Microsoft Office Excel using a plugin provided by national instruments located at http://www.ni.com/example/27944/en/.

### Control of Bioflo110 using LabVIEW

**Figure 1:**
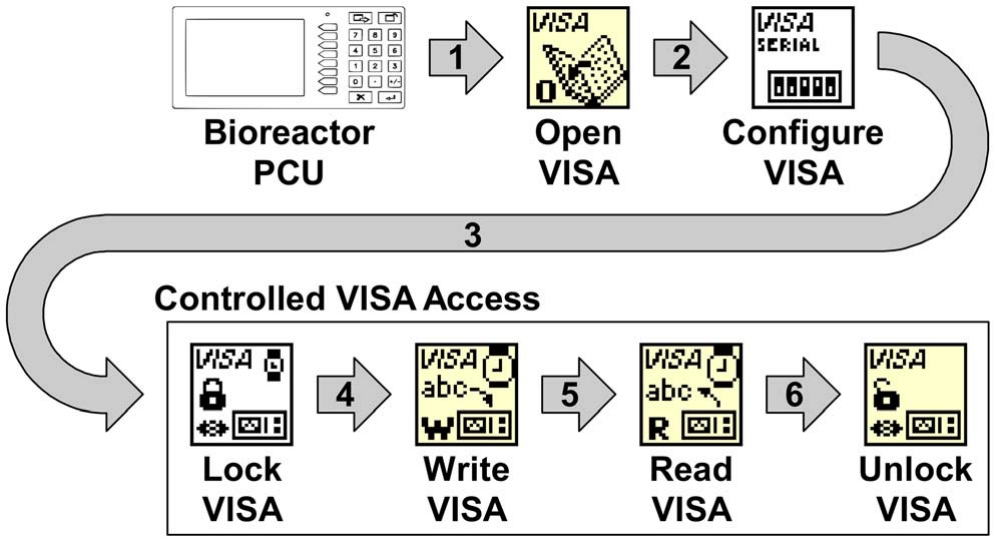
Bioreactor Communication. The PCU communicates with the computer using Labview Virtual Instrument Software Architecture (VISA). A VISA resource is created with the PCU serial port specified by the user at run time. The VISA resource is used to open and configure a connection between the PCU and PC (1&2). A separate VISA resource is created for each of the four Bioflo110 reactors. To avoid communication errors caused by race conditions, the “VISA Lock” function is used by each control system to obtain sole access to the serial port (3). The VISA resource is used to write messages using the “VISA Write” function (4), and read responses using the “Read VISA” function (5). After finishing communication with the PCU the “VISA Unlock” function is used to release access to the serial port.

The AFS communications protocol was implemented using LabVIEW's Virtual Instrument Software Architecture (VISA). VISA is a high level application programming interface that calls into low-level instrument drivers [13]. This architecture facilitates control of diverse instruments through a single interface. Using the “VISA Open” function a live connection to the PCU is created (Figure 1). The “VISA Configure Serial Port” then formats the serial connection with the appropriate baud rate, data bits, stop bits and parity specified in the Bioflo110 Guide to Operations. Sending messages to the PCU is achieved with the “VISA Write” function. The messages are formatted as character strings with numeric values in American Standard Code for Information Interchange (ASCII) decimal format. PCU responses are read through the use of the “VISA Read” function. The PCU messages are ASCII character strings formatted according to the AFS communications protocol.

### Bioreactor VI

**Figure 2:**
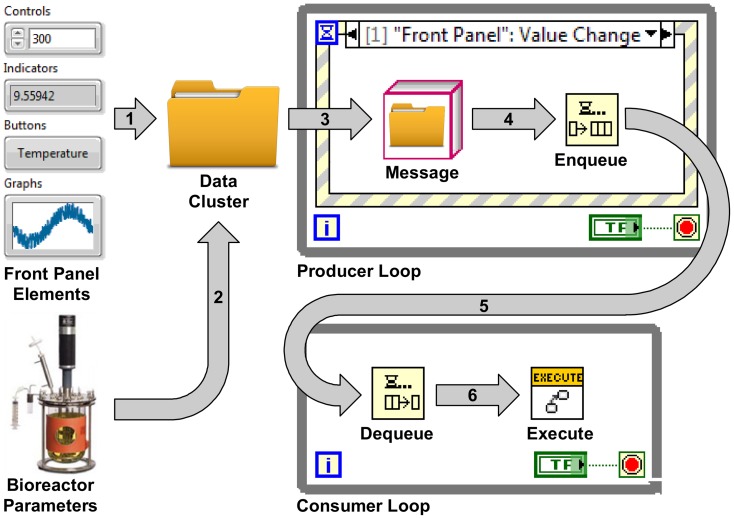
Bioreactor VI Block Diagram. The Labview block diagram execution flow is left to right in a parallel fashion as computers processor resources become available. At run time, the bioreactor program is passed a data value reference (DVR) to the shared bioreactor parameters. The program bundles the bioreactor parameters in a data cluster with references to all front panel indicators and controls (1 & 2). In response to a front panel transitions, a message is created with the data cluster and action instructions (3) and enqueued in a massage queue (4) that is shared with the consumer loop. The consumer loop waits for (5), and dequeues messages (6),executing the instructed actions with the provided data cluster.

**Figure 3:**
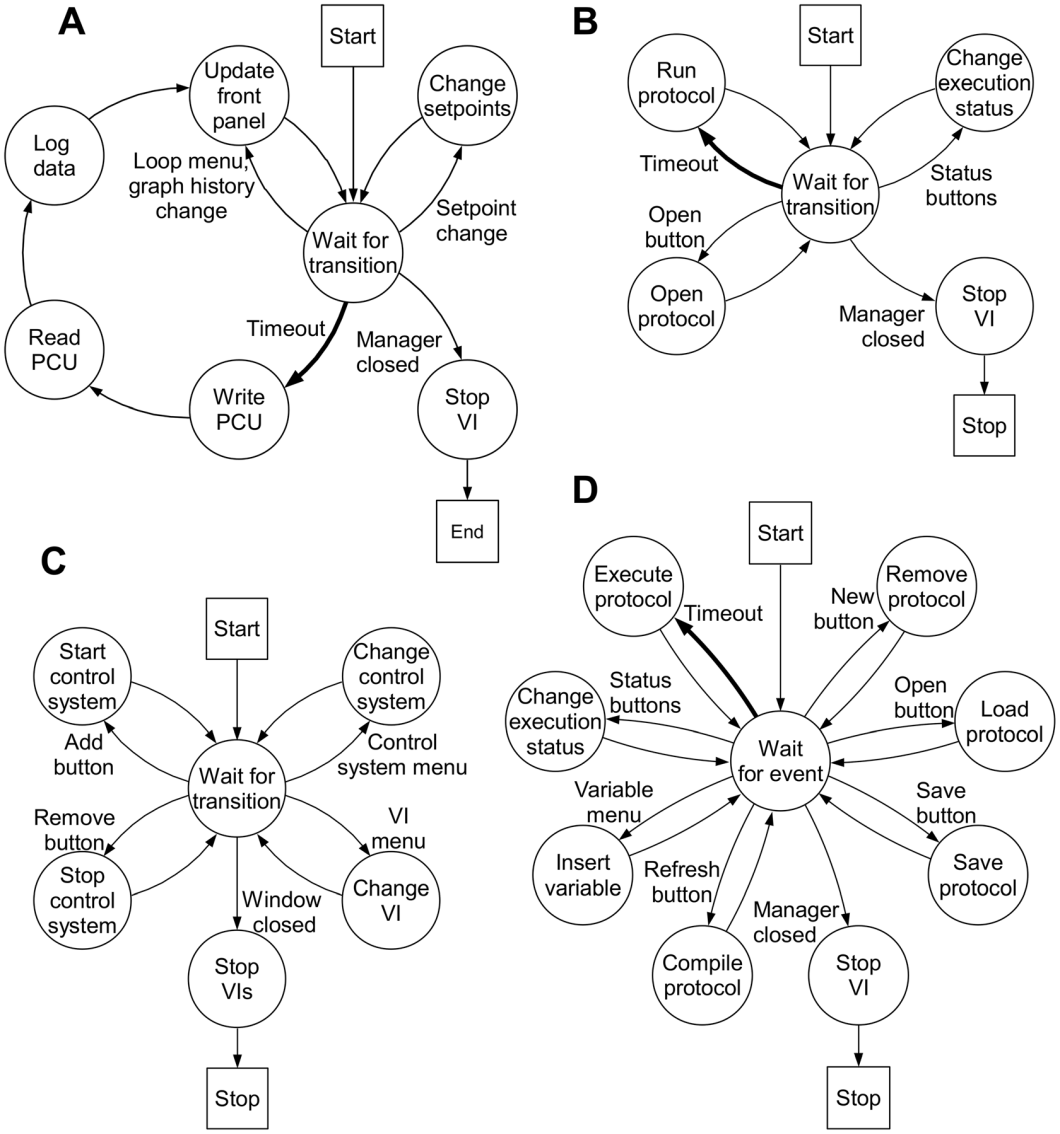
Logic Diagrams. Visual representation of VI transitions and actions. Transitions and actions are represented by arrows and circles. The starting and ending states are represented by squares. Program execution flows from the start to end in the direction of the arrows. Transition timeouts, shown in bold, are the only transition that are not user prompted and occur after a period of time elapsed without transitions. **3A Bioreactor VI:** Transition timeouts start a series of actions that writes bioreactor setpoints, reads bioreactor values and outputs, logs data, and updates the front panel. Loop name or graph history value changes update the front panel, while a setpoint value change updates the control loop setpoints. **3B Time Protocol VI:** Execution status value changes alter the status to play, pause or stop. The play status allows execution of the loaded protocol. The pause status suspends protocol execution. The stop status restarts the protocol and suspends protocol execution. **3C Manager VI:** An add/remove control system button value change starts/stops control systems. A control system menu value change modifies the control system VIs listed in the VI menu. A VI menu value change modifies the VI displayed in the subpanel. **3B Python Protocol** A: new button value change clears the protocol path, protocol script and compiler output on the front panel. An open button value change prompts the user to select a protocol to load. The selected protocol text and path are placed in the protocol script and protocol path elements on the front panel. Bioreactor parameters used in protocol creation/modification are imported to the protocol script by selecting items from the variable menu.

A main bioreactor VI was developed to control the Bioflo110 hardware and perform data logging. The front panel of the bioreactor VI provides a graphical user interface to visualize and adjust control loop outputs, setpoints, and current values. For each control loop, the output, setpoint, and value are updated at set intervals and graphed as a function of time. Data is simultaneously logged in LabVIEWs TDMS file format, which is a digital data format directly accessible through Microsoft Excel for later analysis. To enhance data survey within the bioreactor VI, the history lengths of graphed parameters were made individually adjustable. The block diagram of the bioreactor VI was designed using the QSM-PC architecture. The QSM-PC was used to decouple front panel user inputs, which are referred to as transitions, from code execution on the block diagram, referred to as actions. Actions, such as hardware communication, generally have slow execution times compared to the rate at which transitions can be generated. If transitions and actions are coupled to the same process, VI responsiveness to transitions only occurs after the execution of an action is finished. By decoupling transitions from actions the bioreactor VI's responsiveness to transitions is not hindered by the execution time of actions. Decoupling was achieved with two parallel processes, called the producer and the consumer loop. The producer loop maintains VI responsiveness to transitions by delegating actions to the consumer loop with shared access to a queue of messages (Figure 2). Messages contain instructions and data that are needed for the execution of actions (Figure 2). Messages are generated and enqueued by the event structure, which is contained within the producer loop. An event structure can contain multiple execution cases that are uniquely linked to transitions. Each case generates and enqueues messages with different action instructions. After enqueueing a message, the event structure returns to a default state where it waits for the next transition. The consumer loop removes messages from the shared queue and executes the instructed actions (Figure 2). This behavior continues iteratively to empty the queue. Once the queue is emptied, the consumer loop returns to a default state and waits for more messages to appear in the queue. Figure 3A shows all the transitions and actions used in the bioreactor program. The QSM-PC architecture scales well within LabVIEW because each VI encapsulates its own consumer and producer loop, allowing for the parallel operation of all the VIs and subVIs.

### Protocol execution

To be able to run experiments that require automated changes in the control parameters, the software was designed to include protocol execution. Two separate VIs, the Time Protocol VI and the Python Protocol VI, interact with the bioreactor VI, including all protocol functionality. By implementing protocols as separate VIs, protocol VI development does not require any modifications to the existing code. This modular solution is well-suited for development of multi-process systems because interaction between software routines is minimized. The protocol VI block diagrams utilized the same QSM-PC architecture as the bioreactor VI with different transitions and actions (Figure 3B&C). The reuse of the bioreactor VI structure increased protocol VI functionality and reduced development time.

### Time Protocol VI

**Figure 4:**
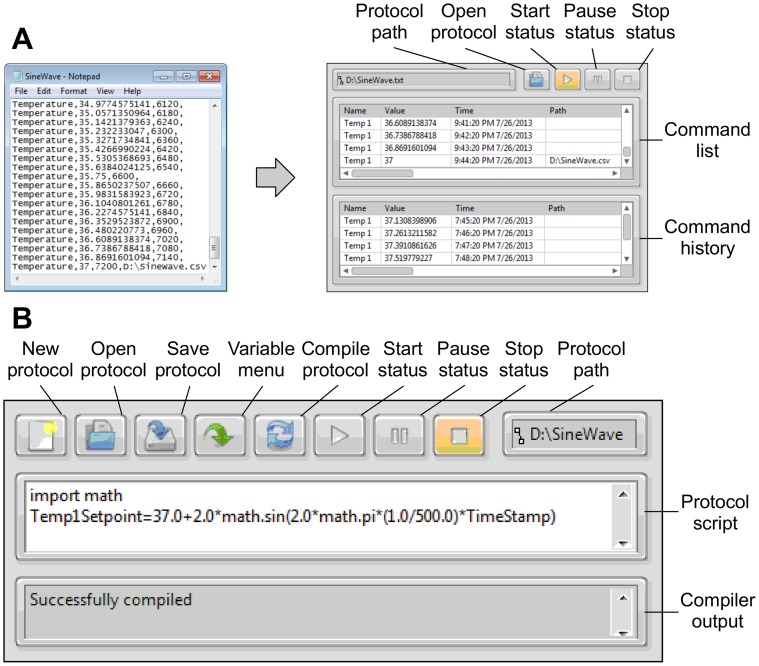
Protocol VIs: 4A Time Protocol. Protocol command parameters are variable name, update value, execution time and optional parameter, load path. The VI parses the selected CSV file and stores its contents in the command history indicator. When running, the Protocol VI parses commands for time parameters that have been surpassed by the present time. These commands are removed from the command list and executed by assigning the specified variable with the update value. If there is a load path parameter present, the protocol located at that path is loaded and appended to the commands in the command list. After a command has been executed it is stored in the command history indicator, which displays the last 1000 commands executed. **4B Python Protocol** The python protocol VI uses an open source library called LabPython to execute script protocols through an interface with the Python scripting language. Protocols can be loaded by selecting the open button and developed in the protocol script control using variable inserted from the variable menu.

The time protocol VI modifies control loop setpoints at specific times according to a protocol script. Protocol scripts are command lines that contain: 1) execution time; 2) variable name; and 3) setpoint value. Protocol files can be opened from protocol command lines as well, allowing for the recurrent operation of a protocol, or the concurrent execution of additional protocols. Each Bioflo control setpoint that is available through the VISA interface can be modified using the protocol VI. The protocol scripts are stored in comma separated value (CSV) files which contain a protocol command in each line. The CSV file format was chosen to facilitate protocol editing in Microsoft Excel. Scripts are loaded and executed with the toolbar buttons located on the front panel of the protocol VI (Figure 4A). When a protocol is loaded the VI parses the CSV file, stores its contents in the command list, and displays the file path in the toolbar. Protocol execution is started, paused, and stopped with three buttons that define the three mutually exclusive operation states the protocol VI can be in. By transitioning to the “start” state the protocol time parameters are converted from relative times to absolute times, and the protocol VI executes commands from the command list. If a load protocol command is encountered, the protocol is loaded, and integrated into the command list. After a command is executed it is moved to the command history indicator. Programmatic loading of protocols is especially useful for cyclic operations. As an example of a protocol that implements a cyclic operation the SineWave.csv file was created (Supplement S2). The SineWave.csv file contains a series of commands that sinusoidally oscillates the temperature of the bioreactor over one period. The last command in the protocol contains the load command for itself. This prompts the protocol VI to load another period of the oscillation into memory.

### Python Protocol VI

The python protocol VI uses an open source library called LabPython to interface with the Python scripting language. Python is an open source object orient code with a syntax that is designed to be highly readable. By utilizing the Python language conditional logic can be implemented in protocols within the same familiar, stable environment. The front panel of the python protocol VI (Figure 4B) provides a protocol development environment capable of compiling, opening and saving protocols as wells as executing protocols. The python protocol was tested by duplicating the functionality of the SineWave.csv time protocol. This was achieved with only two lines script code (Supplement S3). While the python protocol VI allows users familiar with text based code to develop protocols that are more complex than that can be achieved with the time protocol VI, the python protocol requires understanding of the python language.

### Data access model structure

**Figure 5:**
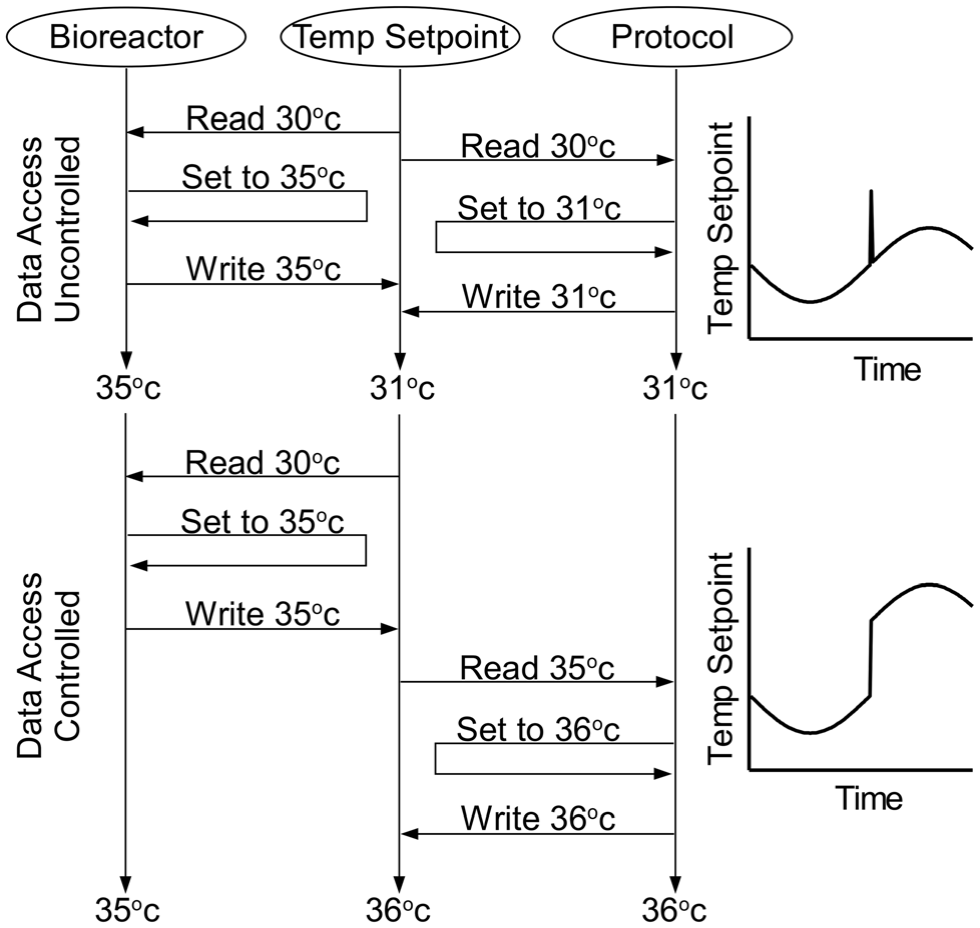
Race Condition. A race condition occurs when two processes share asynchronous access to a variable, which can lead to unpredictable changes in a variable if two processes race each other to modify the variable value. A GUI allows users to change the temperature setpoint value while the protocol program sinusoidally oscillates the temperature setpoint value. A 5°C increase in the oscillation offset from the user could be nullified by the protocol program if its operations on the variable were concurrent with the bioreactor operations (uncontrolled data access). Synchronous access to the variable guaranties that the bioreactor program's change will not be nullified by the protocol program (controlled data access).

**Figure 6:**
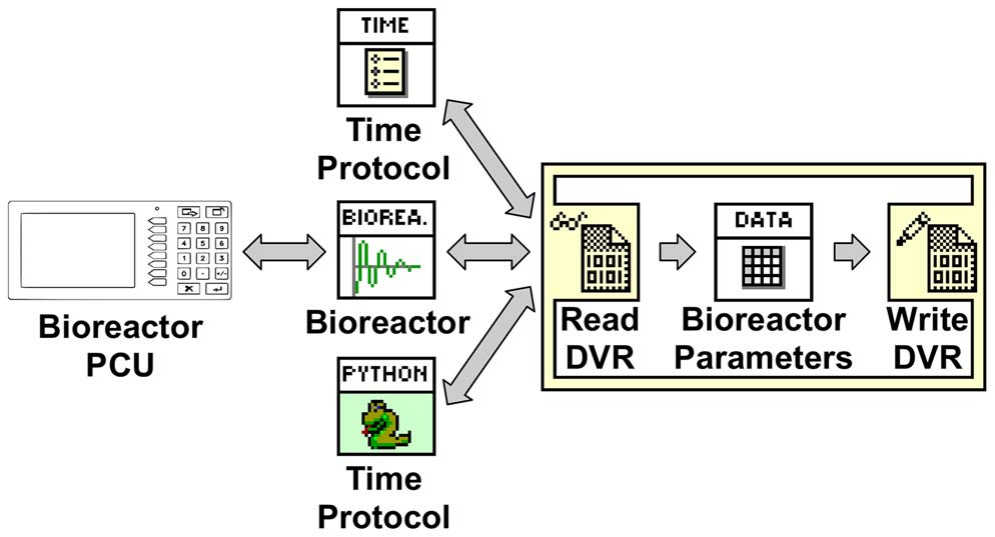
Data Structure. Bioreactor parameters access is synchronized by providing the bioreactor, time protocol and python protocol VIs with a data value reference (DVR) to the parameters. The DVR is used to access the bioreactor parameters through an in place element structure. The in place element structure performs operations on the bioreactor parameters without creating a copy in memory. This data structure enables the data exchanged with the bioreactor PCU to be shared with the protocol VIs without causing race conditions.

The implementation of protocols, such as the SineWave.csv, required access to reactor parameters. This was achieved by storing reactor parameters in a shared data source. The bioreactor VI and protocol VIs read from, and write to, this shared data source. Access to the data source was regulated to ensure that all processes only access current information. Utilization of outdated information, such as data that is in the process of being updated, can lead to intermittent errors caused by ‘*race conditions*’. Race conditions occur when two parallel processes race each other to modify a shared resource. Occasionally processes that utilize outdated data can overwrite values, effectively reverting a value to an earlier state. For instance, if data source access is not regulated, a race condition could occur in the temperature setpoint that is shared between the bioreactor and protocol VIs (Figure 5). If the operator raises the temperature setpoint *after* the protocol reads the temperature setpoint, but *before* the protocol saves the new setpoint, the temperature setpoint will fall back to its initial value following a short spike (Figure 5). Data access control was implemented using a lock, read, modify, write, and unlock sequence (Figure 6). This sequence locks the data source, which prevents access by other parallel VIs (i.e. the VI has sole access to the data source). The VI performs all operation on the data source during this time period. After saving the modifications, the data source is unlocked, which releases the data source to other VIs. All VIs attempting to access a locked data source are queued and given access rights on a - first in first out - basis. All VIs access the shared data source with a data value reference (DVR), which is passed to each VI at run time.

### Multiple bioreactors/shared hardware

**Figure 7:**
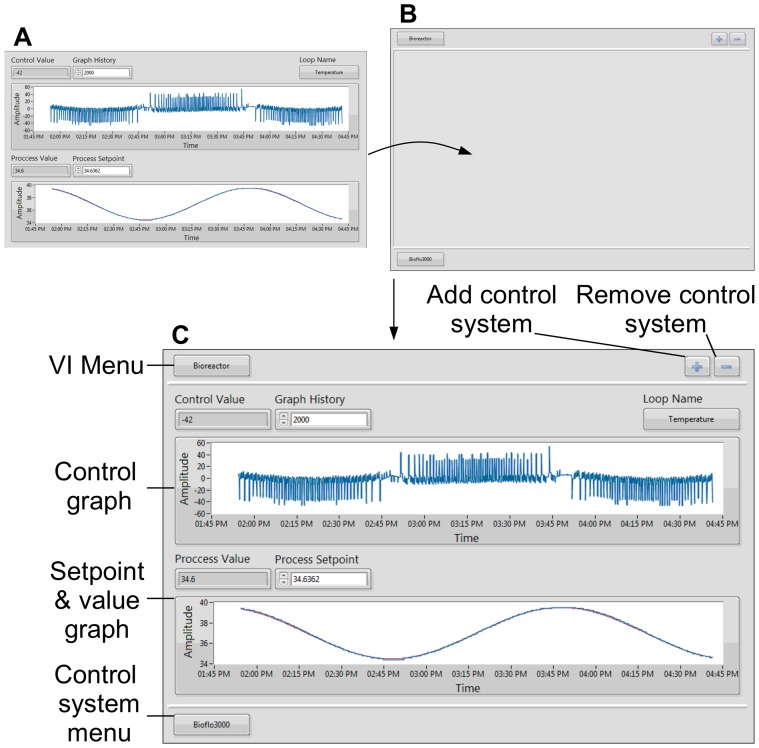
Bioreactor & Manager VI: 7A Bioreactor VI: The bioreactor VI front panel provides an interface for monitoring and controlling the Bioflo reactor. The control value, process value and process setpoint are displayed with numerical indicators and graphs. The numerical indicators display the most recent values, while the graphs display the values over a period of time specified by the graph history control. The process setpoint is user controllable, and displayed graphically with a red line. The control loop in view is selected in the loop name menu. **7B Manager VI:** The manager VI provides a single interface for all control system VIs through a font panel indicator called a subpanel. The subpanel displays the front panel of others VIs, which are selectable through the VI and control system menus. The control system's VIs in view are selected through the control system menu. Each control system has a bioreactor, time protocol and python protocol VI. The VI in view for a given control system is selected through the VI menu. The manager VI also contains the ability to dynamically start and stop control systems though the “add and subtract control system” buttons. **7C Manager & Bioreactor VI:** In this example the manager VI is displaying the temperature control loop of the bioreactor VI from a Bioflo3000 control system. The graphs show the response of the Bioflo3000 to the execution of the SineWave.csv time protocol.

The BioFlo 110 PCU is capable of controlling up to four bioreactor units concurrently. To utilize this functionality, the ability to control multiple bioreactors from a single PCU was added to the software. Individual bioreactor control was implemented by using independent copies of the bioreactor and protocol VIs for each reactor, which allows for custom control of each bioreactor. To interact with each bioreactor program through a single user interface, a manager VI was introduced to encapsulate all VIs (Figure 7). The manager VI block diagram contains the same QSM-PC architecture used in the bioreactor VI and protocol VI, but contains different transitions and actions (Figure 3D). The front panel of the manager VI contains a subpanel that displays the front panel of the bioreactor and protocol VIs. The VI displayed in the subpanel is selectable through the VI and control system menus (Figure 7). The control system menu selects which bioreactor control systems is displayed, while the VI menu selects between python protocol VI, time protocol VI and bioreactor VI. The manager VI front panel also contains controls for adding and removing a bioreactor control system. This feature enabled the start and stop of additional bioreactors. By closing the manager VI, a system wide stop condition is issued to all bioreactor and protocol VIs.

Each bioreactor VI was given access to the PCU to allow the bioreactor VIs to operate independently. Similar to the data source access, the PCU access was regulated to prevent race conditions. Uncontrolled PCU access could cause a race condition in which one bioreactor VI could revive old data that was modified by another bioreactor VI, but not yet stored. PCU access control was implemented by locking the VISA resource before writing and reading operations (Figure 1). By controlling access to the serial port associated with a VISA resource, PCU access was shared between bioreactor VIs without sharing VISA resources. This was necessary because a bioreactor VI's VISA resource is created and destroyed when its control system is added and removed by the Manager VI.

The precautions taken to decouple the control of individual Bioflo110 reactors enabled the simultaneous control of multiple Bioflo110 PCUs as well as other Bioflo family bioreactors that use the AFS communications protocol. This was demonstrated by controlling multiple Bioflo110 bioreactors through a single PCU, while simultaneously controlling a Bioflo3000 bioreactor. This method of hardware sharing can be replicated amongst plugins with axillary hardware, adding functionality to the control system. By relegating the control of auxiliary hardware to plugins, and controlling the access of hardware amongst plugins, this additional functionality comes with little risk to the stability of the main bioreactor VIs.

### Conclusion

Research bioreactors are instrumental in creating a carefully controlled laboratory environment for experimentation on microbes and cell cultures. Further enhancement of the control functionality of research reactors is a natural extension of a reactor's core functionality. Yet, commercially available bioreactors are not easily interfaced with auxiliary hardware, or controlled with custom software. This work addressed these limitations by implementing a QSM-PC software architecture with a plugin interface in LabVIEW software to provide an open source software package for the control of bioreactors of the New Brunswick Scientific Bioflo product family. The software package enables process parameter control, provides an interface for process monitoring, implements data logging and creates the capability to execute user defined protocols. A single manager VI encapsulates all functionality and allows for parallel control of multiple Bioflo bioreactor control systems. This software was tested with the Bioflo110 and Bioflo3000 control system by executing a simple protocol that sinusoidally oscillates the temperature setpoint. The presented opens source software is a flexible solution to help unlock the potential of bioreactor control in research. This software can be adapted to control other bioreactors, but is primarily intended to alleviate the investment in time and resources needed to extend the functionality of commercially available Bioflo bioreactor systems.

## Supporting Information

Supplement S1 **supplement.pdf Connection to, and communication with the Bioflo reactor.**
(PDF)

Supplement S2 **SineWave.csv. Example script for the Time VI protocol.**
(CSV)

Supplement S3 **SineWave.txt. Example script for the Python VI protocol.**
(TXT)
