# Supplementary material for: Open Source Software to Control Bioflo Bioreactors
Source: PLoS One. 2014 Mar 25;9(3):e92108. doi: 10.1371/journal.pone.0092108 (PMC3965399; doi:10.1371/journal.pone.0092108)
Supplement: Supplement S1 — supplement.pdf Connection to, and communication with the Bioflo reactor. (PDF) [file pone.0092108.s001.pdf]

Open Source Software to Control Bioflo Bioreactors

David A Burdge and Igor GL Libourel

SUPPLEMENT

Figure 1: Patch cable to connect a Bioflo machine to a serial port

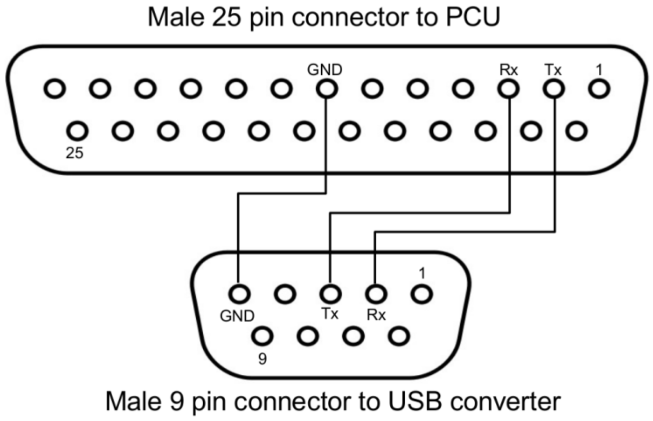

The Bioflo110 primary control unit (PCU) is equipped with a 25 pin connector used for communicating with a personal computer. The connector was configured for RS-232 communication standard. The 25 pin connector was converted to a 9 pin connector: the Tx, Rx and GND pins of the 25 pin connector were soldered to the Rx, Tx and GND pins of the 9 pin connector.

Figure 2: Communication command structure

I **Request Message**

Type	Format	Response (Header)
Get Loop Names	(MD#)RA(CR)	Loop(Sp)(Sp)(Sp)(Sp)(Sp)
Get Current Values	(MD#)RC(CR)	Cur:Value
Get Setpoints	(MD#)RD(CR)	OP Points
Get Controller Outputs	(MD#)RE(CR)	Output(Sp)%(Sp)
Get Control Modes	(MD#)RJ(CR)	Control(Sp)(Sp)
Get Units	(MD#)RL(CR)	Main(Sp)Unit

II **Command Message**

Type	Format (Header)	Response
Change Setpoints	MS(Sp)(Sp)(Sp)(Sp)(Sp)(Sp)(Sp)	(MD#)ACK
Change Controller Output	MO(Sp)(Sp)(Sp)(Sp)(Sp)(Sp)(Sp)	(MD#)ACK

III **Request Response & Command Message Formats**

(MD#)	Header 9 chars	Field 1 6 chars	(Sp)	…	Field 10 6 chars	(Sp)	(CR)

(MD#) = Multidrop ID Number, (CR) = Carriage Return, (Sp) = Space,
ACK = Acknowledgement character

The AFS communications protocol uses a master-slave model where the PC is the master and the PCU is the slave. All communications are initiated by the PC, requesting a response from the PCU. Messages to/from the PCU are preceded by a multidrop number that specifics the vessel control system. The PC sends two types of messages: requests and commands. The request messages prompt the PCU to respond with specific information. A list of request types are shown in the first column of table I. Request message formats are shown in the second column of table I. The PCU responds to request messages with character strings formatted according to table III with response headers shown in third column of table I. The command messages prompt the PCU to change a setpoint or controller output value. The massages are formatted according to table III with format headers shown in the second column of table II.

Protocol execution:

Detail of an example script for the **Time Protocol VI** (full executable script is included as an additional supplement).

Temp 1	37.13084	60
Temp 1	37.26132	120
Temp 1	37.39109	180
Temp 1	37.51978	240
Temp 1	37.64705	300
Temp 1	37.77254	360
Temp 1	37.89592	420
Temp 1	38.01684	480
Temp 1	38.13498	540
Temp 1	38.25	600
Temp 1	38.3616	660
Temp 1	38.46946	720
Temp 1	38.5733	780
Temp 1	38.67283	840
Temp 1	38.76777	900
Temp 1	38.85786	960
Temp 1	38.94286	1020
Temp 1	39.02254	1080
Temp 1	39.09668	1140
Temp 1	39.16506	1200
Temp 1	39.22752	1260
Temp 1	39.28386	1320
Temp 1	39.33395	1380
Temp 1	39.37764	1440
Temp 1	39.41481	1500

Each line contains: 1) the controlled parameter; 2) the parameter setpoint, and; 3) the execution time relative to the start of protocol execution. All parameters, including from dynamically loaded plugins can be programmed in this fashion.

Example script for the **Python Protocol VI** (full executable script is included as an additional supplement).

```
import math
TempSetpoint=37.0+2.5*math.sin(2.0*math.pi*(CurrentTime-StartTime)*(1.0/7200.0))
```

The python protocol VI uses an open source library called LabPython to interface with the Python scripting language. By utilizing the Python language conditional logic can be implemented in protocols.
